# Impact of pneumococcal conjugate vaccine uptake on childhood pneumonia mortality across income levels in Brazil, Colombia, and Peru

**DOI:** 10.12688/gatesopenres.13187.1

**Published:** 2020-09-22

**Authors:** Kayoko Shioda, Cristiana M. Toscano, Maria Tereza Valenzuela, William Valdez Huarcaya, Joshua L. Warren, Daniel M. Weinberger, Lucia H. de Oliveira

**Affiliations:** 1 Department of Epidemiology of Microbial Diseases, Yale School of Public Health, New Haven, CT, USA; 2 Department of Collective Health, Institute of Tropical Pathology and Public Health (IPTSP), Federal University of Goiás (UFG), Goiânia, Goiás, Brazil; 3 Department of Epidemiology and Public Health, Universidad de Los Andes, Santiago, Chile; 4 Universidad Peruana de Ciencias Aplicadas, Lima, Peru; 5 Department of Biostatistics, Yale School of Public Health, New Haven, CT, USA; 6 Comprehensive Family Immunization Unit/FPL, Pan American Health Organization, World Health Organization, District of Columbia, USA

**Keywords:** Pneumococcal conjugate vaccine, vaccine evaluation, vaccine coverage, vaccine uptake, Brazil, Colombia, Peru, income level, childhood pneumonia mortality

## Abstract

**Background:** Pneumococcal conjugate vaccines (PCVs) have prevented deaths due to pneumonia among children. The effect may differ between higher- and lower-income populations due to various factors, such as differences in the distribution of pneumococcal serotypes, healthcare access, and PCV uptake. This study aims to evaluate an association between increasing PCV coverage and population-level declines in death due to pneumonia and its variation by socioeconomic status of subnational regions.

**Methods: **We analyzed municipality-level mortality data from 2005 and 2015 for children aged 2-23 months in Brazil, Colombia, and Peru. We fit Poisson regression models to estimate the relationship between changes in PCV uptake and deaths due to all-cause pneumonia among subnational regions with different income levels. We controlled for changes unrelated to PCV by using data on non-respiratory deaths over time.

**Results: **Uptake of the third dose of PCV varied across subnational regions and was higher in high-income regions. Higher uptake of PCV was associated with larger declines in pneumonia mortality. This association did not differ by income level of the region in Brazil and Colombia. In Peru, low-income regions observed larger declines in pneumonia deaths, but there was large uncertainty in the difference between the low- and high-income regions. We estimated that, with 90% coverage, there would be 4-38% declines in all-cause pneumonia mortality across income levels and countries.

**Conclusions: **Regions with higher PCV coverage experienced larger declines in pneumonia deaths, regardless of the income level. Having more reliable data on mortality records and vaccine uptake would improve the reliability of vaccine impact estimates.

## Introduction

Vaccination programs targeting pneumococcus are now established globally. Pneumococcal conjugate vaccines (PCVs) have effectively reduced the burden of disease and death due to pneumonia and other severe pneumococcal infections (e.g., septicemia, meningitis) ^1– 7^. For instance, national-level analyses found that estimated declines in pneumonia mortality among children 2–11 months of age were 8% (95% credible interval (CrI): 2 to 14%) in Brazil, 14% (-5 to 35%) in Colombia, and 36% (17 to 44%) in Peru ^7^. The impact of PCVs on preventing deaths due to pneumonia could differ between higher- and lower-income populations. Differences in the etiology of pneumonia, the distribution of pneumococcal serotypes, and access to healthcare can influence the proportion of deaths that are preventable. Variations in vaccine uptake can also influence the overall reduction.

The countries in the Latin America and Caribbean region are ideal for conducting evaluations of the impact of PCVs. Countries in this region were among the first lower- and middle-income countries to introduce PCVs. The strong health and mortality data systems in these countries make it possible to perform detailed vaccine evaluation studies at a subnational level and to evaluate how variations in vaccine uptake relate to changes in rates of pneumonia deaths. The countries also have a wide variation in socioeconomic status at a subnational level, which provides a unique opportunity to compare the impact of PCV by income level. Therefore, this study aims to evaluate how the association between increasing vaccine uptake and population-level declines in death due to pneumonia varies by socioeconomic status in Brazil, Colombia, and Peru.

## Methods

### Mortality data and stratification

In a previous study, we evaluated the population-level impact of PCVs against pneumonia mortality at the national level in ten Latin American and Caribbean countries ^7^. Among the 10 countries included in the national-level study, Brazil, Colombia, and Peru had subnational data on mortality, socioeconomic status, and vaccine coverage with a high spatial resolution, which enabled us to conduct this analysis. Mortality data were obtained from the National Mortality Information Systems. Both primary and non-primary causes of deaths were recorded for Brazil and Peru, while only primary causes were available for Colombia. The cause of death was classified using the International Statistical Classification of Diseases and Related Health Problems tenth revision (ICD-10) codes. Each country conducted standardized data cleaning and quality control ^8^. We extracted mortality data for children at the municipality level that were available during the study period (2005-2015). The quality of mortality data was assessed and reported in the previous study ^7^. Durations of pre- and post-PCV periods for each country are described in Table 1.

**Table 1.** Descriptive statistics for Brazil, Colombia, and Peru.

	Brazil	Colombia	Peru
Pre-PCV period	January 2005 - February 2010 (5 years and 2 months)	January 2005 - October 2011 (6 years and 10 months)	January 2005 - July 2009 (4 years and 7 months)
Post-PCV period	March 2010 - December 2015 (5 years and 10 months)	November 2011 - December 2015 (4 years and 2 months)	August 2009 - December 2014 (5 years and 5 months)
Total number of J12-18 among children 2–23 months of age in the study period (coded as primary or non-primary causes of death) ^1^	16,303	4700 (primary only)	6854
Total number of J12-18 among children 2–23 months of age in the study period (coded as primary cause of death) ^1^	35,259	4700	5818
Weighted average of PCV third dose coverage in the last year of the study period	91.7% in 2015	91.1% in 2015	81.8% in 2014

^1^These numbers are before filtering out regions with sparse data. The study periods include both pre- and post-PCV periods. Abbreviation: PCV, pneumococcal conjugate vaccine.

An outcome of our analysis was all-cause pneumonia deaths, defined as having an ICD-10 code in the range of J12-J18. For Brazil and Peru, we used J12-J18 recorded as either primary or non-primary causes of death as the outcome in the main analysis, and used J12-J18 recorded as the primary cause of deaths as the outcome in the sensitivity analysis. For Colombia, we used J12-J18 recorded as the primary cause of death as the outcome, as they did not have data on non-primary causes of death.

We analyzed data for children 2–23 months of age, as our previous national-level analysis did not detect any changes in pneumonia mortality among older children (24–59 months of age) following the introduction of PCV in any of the ten countries we analyzed ^7^. Children under two months of age were not included in the analysis for both biological and practical reasons ^7^.

For Brazil and Colombia, we classified municipalities into three income levels (low, medium, and high) using the Gross Domestic Product (GDP) per capita for the year of PCV introduction. For Peru, these three categories of the income level were created based on a five-level income level indicator, reflecting the per capita income in the year of PCV introduction. This indicator is obtained from household surveys and is based on quintiles of household per capita expenditure as it relates to the costs of a “basic consumption basket.” ^9, 10^ These income categories were defined for each country separately and cannot be directly compared across countries. We then created subnational regions by grouping municipalities based on these three income-level categories in each of the departments (n=32 in Colombia and n=25 in Peru) or states (n=27 in Brazil). For example, Peru had 75 possible combinations of departments and income levels; however, there were only 68 subnational regions simply because some strata did not exist (e.g., no low-income regions in Lima). Furthermore, we excluded subnational regions from the analysis if there were less than ten all-cause pneumonia mortality per year on average. After this exclusion, we had 49 subnational regions in Brazil (11 low-, 25 medium- and 13 high-income regions), 16 in Colombia (12 medium- and four high-income regions), and 23 in Peru (five low-, 10 medium-, and eight high-income regions) in the final analysis.

### PCV coverage data

Brazil introduced the 10-valent PCV with a 3+1 schedule (2, 4, and 6 months + 12–18 months of age) in March 2010. Colombia introduced the 10-valent PCV in November 2011 and used a 2+1 schedule (2 and 4 months + 12 months of age). Peru introduced the 7-valent PCV with the 2+1 schedule in August 2009 (2 and 4 months + 12 months of age), and switched to the 10-valent PCV with the 2+1 schedule in December 2011.

Data on the PCV third dose coverage (i.e., last primary dose for Brazil and booster dose for Colombia and Peru) by year were obtained from the National Immunization Programs at the municipality level in all three countries. For Colombia and Peru, we first conducted linear interpolation for missing coverage data, if there were any, in each municipality. We then calculated a weighted average of the coverage in each subnational region. We used the population size of each municipality as a weight for Brazil and Colombia. For Peru, a total number of deaths during the study period in each municipality was used as a weight, as municipality-level population data were not available.

### Statistical analysis

To estimate changes in pneumonia mortality among children after the introduction of PCVs, we fit the following Poisson regression model to the data from each country separately such that

$$Y_{i,t}|\lambda_{i,t}∼\text{Poisson}(\lambda_{it})\;where$$

$$ln(\lambda_{i,t})=\beta_{0i}+\beta_{1i}{\text{x}}_{i,t}+\theta_{i}{\text{v}}_{i,t}+\phi_{i,t}.$$

The outcome, *Y _i,t_*, is the number of all-cause pneumonia deaths in subnational region *i* in year *t*. To control for unmeasured temporal factors affecting pneumonia mortality, we included all-cause deaths other than those caused by respiratory illness as a covariate in the model (x *_i,t_*) where *β*_1 *i*_ represents the region-specific regression parameter that describes the association between this covariate and deaths and *β*_0 *i*_ is the region-specific intercept. To account for potential overdispersion and unexplained correlation in deaths across time, we included an observation-level random effect, *ϕ*_*i,t*_, that follows a first order autoregressive process. The weighted average of the PCV third dose coverage in subnational region *i* in year *t* is given as v _*i,t*_ and the region-specific vaccine effect, *θ _i_*, is modeled as a function of region-level socioeconomic status such that

$$\theta_{i}=\gamma_{1}I_{low_{i}}+\gamma_{2}I_{med_{i}}+\gamma_{3}I_{high_{i}}+\eta_{i}$$

where $I_{low_{i}}$, $I_{med_{i}}$, and $I_{high_{i}}$ are indicator variables for the income level. The remaining variation in *θ _i_* that is not explained by the effect of income level is captured by *η _i_* which are modeled using independent Gaussian distributions with zero mean and shared variance parameter.

Posterior medians were used as point estimates and the 95% highest density CrIs were calculated to quantify uncertainty. All analyses were performed in R (Vienna, Austria) ^11^. The aggregated time series data and code can be found in the following GitHub repository: https://github.com/weinbergerlab/PAHO_subnational. A stable copy of the repository is available from https://zenodo.org/badge/latestdoi/291341482. More details on the model, such as the information on prior distributions, can be found in the *Extended data*, Supplementary methods ^12^.

### Estimation of the impact of PCV by income level

We evaluated how the population-level impact of PCV changes by income level in a few different ways. First, we quantified declines in all-cause pneumonia deaths based on the actual coverage in the last year of the study period in each subnational region. Next, to compare the effect of PCVs between regions while holding vaccine uptake constant, we estimated declines in all-cause pneumonia deaths that would be expected with 90% uptake of the third dose of PCV. More details on the calculations are described in the *Extended data*, Supplementary methods ^12^.

## Results

### Variation in vaccine uptake at subnational level in Brazil, Colombia, and Peru

The uptake of PCV increased rapidly within a few years after introduction in all countries, but there was variation by region ( *Extended data*, Figure S1) ^13^. For example, although the average uptake of the third dose was 82% at the national level in the last year of the study period in Peru ( Table 1), average uptake varied from 55% to 100% between regions. Uptake of the third dose was higher in more affluent regions ( *Extended data*, Figure S2) ^13^. For example, in Peru, the median uptake was 67%, 88%, and 90% in low, medium, and high-income regions, respectively, in 2014.

### Declines in all-cause pneumonia mortality by PCV uptake and income level

**Figure 1.**
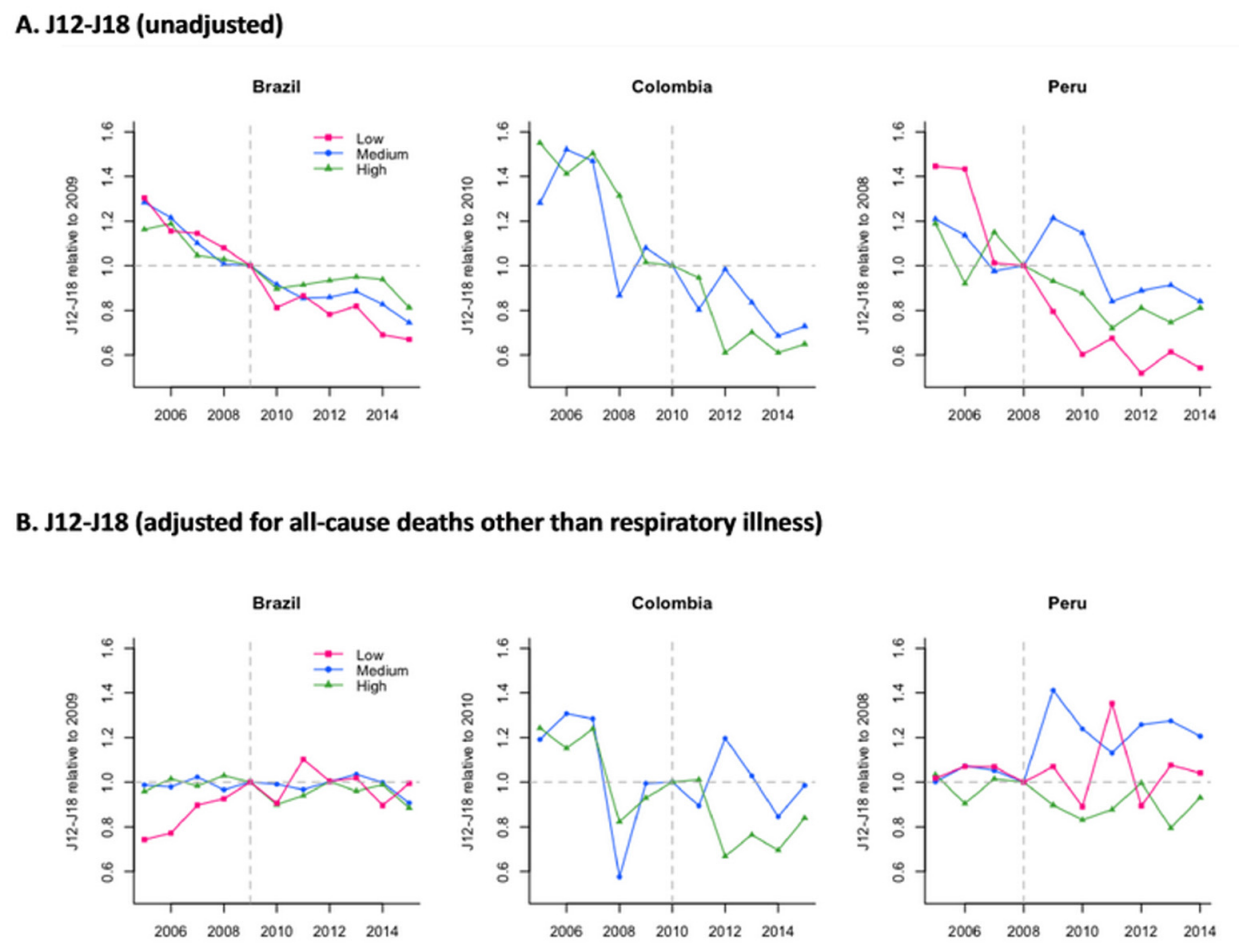
Time series for all-cause pneumonia mortality (ICD-10 code: J12–J18) relative to the counts in the final year of the pre-PCV period by income level among children aged 2–23 months in Brazil, Colombia, and Peru. Pink, blue, and green lines represent estimates for low-, medium-, and high-income subnational regions in each country. Vertical dashed lines represent the final year in the pre-PCV period in each country. Abbreviations: ICD-10, International Statistical Classification of Diseases and Related Health Problems tenth revision; PCV, pneumococcal conjugate vaccine.

**Figure 2.**
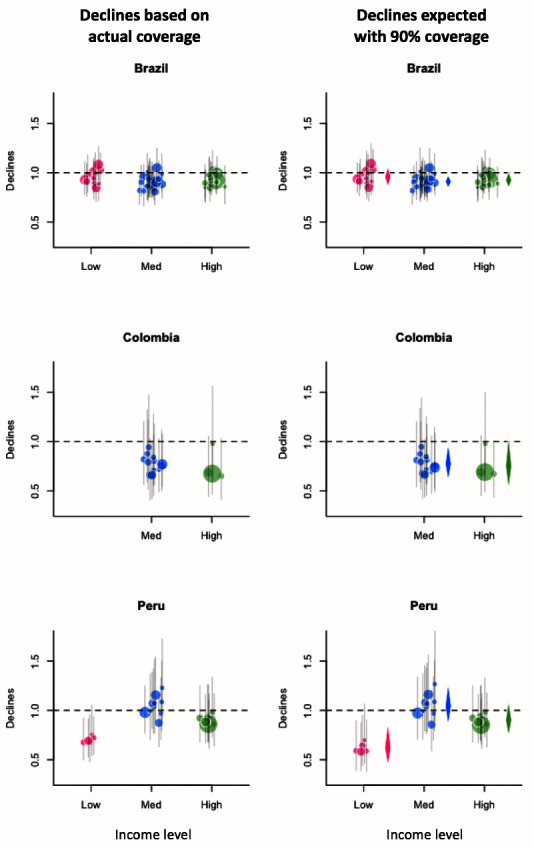
Declines in all-cause pneumonia estimated based on actual coverage in the last year of the study period or with the 90% coverage. Pink, blue, and green bubbles represent the point estimates for low-, medium-, and high-income subnational regions in each country. Grey bars are the 95% CrIs. The size of bubbles is proportional to the number of all-cause pneumonia deaths in the last year of the pre-PCV period. Horizontal dashed line indicates one, meaning that there were no changes in all-cause pneumonia mortality. In panels on the right, diamonds represent the average declines in each income level. Abbreviations: PCV, pneumococcal conjugate vaccine; CrI, credible interval.

**Figure 3.**
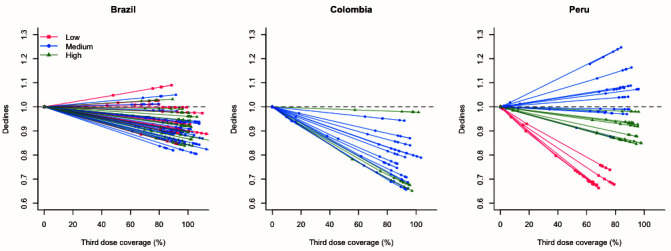
Estimated declines by PCV third dose coverage for each region. Pink, blue, and green dots represent estimates for low-, medium-, and high-income subnational regions in each country. Horizontal dashed line indicates one, meaning that there were no changes in all-cause pneumonia mortality. Abbreviation: PCV, pneumococcal conjugate vaccine.

All-cause pneumonia mortality declined over time across income levels and countries, and these declines started before the introduction of PCV ( Figure 1A). This trend became unclear after adjusting for all-cause mortality other than those caused by respiratory illness ( Figure 1B). In the last year of the study period, most subnational regions across countries observed declines in all-cause pneumonia deaths with large uncertainties, while low-income regions in Peru experienced larger declines ( Figure 2). Higher uptake of the third dose of PCV was associated with additional declines in pneumonia deaths in most of the regions in all three countries ( Figure 3). In Peru, this association was especially strong in low-income regions, while it was modest among high-income regions and not clear among medium-income regions.

With 90% uptake of the vaccine, we estimated declines in all-cause pneumonia would range from 4–38% in low-income regions, -4–23% in medium-income regions, and 8–25% in high-income regions ( Table 2). The estimates for individual regions had a high degree of uncertainty due to small numbers of deaths ( Figure 2). There was no evidence that the effect of the vaccine on pneumonia deaths differed between high- and low-income regions in Brazil and Colombia. In Peru, estimated declines in pneumonia mortality were largest among low-income regions. The estimated decline in pneumonia mortality in low-income regions was 52% (95% CrI: 14%, 90%) and 38% (-2%, 76%) smaller than that in medium-income regions and high-income regions, respectively.

**Table 2.** Average declines in all-cause pneumonia mortality among children aged 2–23 months expected with the 90% coverage of PCV by income level in Brazil, Colombia, and Peru.

Country	Estimated declines (95% CrIs) by income level associated with 90% uptake of third dose of PCV		
	Low	Medium	High
Brazil	0.96 (0.86, 1.06)	0.91 (0.85, 0.97)	0.92 (0.85, 1.01)
Colombia	-	0.77 (0.61, 0.98)	0.75 (0.51, 1.07)
Peru	0.62 (0.44, 0.88)	1.04 (0.85, 1.28)	0.90 (0.74, 1.10)

The outcome for Brazil and Peru is J12-J18 coded as either primary or non-primary causes of death. The outcome for Colombia is J12-J18 coded as the primary cause of death. Abbreviations: PCV, pneumococcal conjugate vaccine; CrI, credible interval.

### Sensitivity analyses

For Brazil and Peru, we repeated the analysis but only included deaths where pneumonia was recorded as the primary cause of death as the outcome variable. This modification did not appreciably change the estimated declines in Brazil, while it increased the estimated impact of PCV in Peru ( *Extended data*, Table S1) ^12^. The discrepancy in Peru was due to changes over time in the proportion of pneumonia deaths recorded as the primary cause vs contributing cause of death ( *Extended data*, Figure S3) ^13^, and this shift exaggerated the estimated impact of PCV when analyzing the primary cause of death only.

## Discussion

Using spatially disaggregated data, we evaluated whether the population-level effect of PCV among children 2–23 months of age varies between income levels in Brazil, Colombia, and Peru. High-income regions had higher coverage of the third dose of PCV. The estimated decline in all-cause pneumonia deaths associated with increasing PCV uptake was similar across income levels in Brazil and Colombia, while there was a larger decline among low-income regions in Peru.

To date, seven post-licensure studies have evaluated the impact of PCV on pneumonia mortality, all of which were conducted in Latin American and Caribbean regions ^1– 7^. These studies used various datasets and methods, and the magnitude of reported effects varied. For example, Diaz *et al.* conducted a nested case-control study in Chile and estimated that pneumonia deaths declined by 71.5% (95% CI: 9.0-91.8%) ^2^. On the other hand, a multi-country study conducted time series analysis and did not find declines in Guyana and Honduras due to large uncertainty ^7^.

A previous study reported larger PCV-associated declines in pneumonia mortality among children in low-income municipalities in Brazil ^6^, while we did not see differences across income levels. That study used three indicators (human development index, children in poverty, and maternal primary education), while we used GDP per capita in the year of PCV introduction. Among children 2–23 months of age, the previous study found the largest decline in low-income regions only when municipalities were grouped based on maternal primary education. Our income classification based on GDP is most closely related to their HDI-based classification. With this HDI category, the previous study found that medium-level regions observed slightly larger declines than low- and high-level regions, which is consistent with our findings for Brazil ( Figure 2 and Table 2).

Data sparsity often becomes an issue when working with subnational data. As death is a rare clinical outcome compared to others (e.g., hospitalizations, outpatient visits), both our outcome (J12–18) and controls (other ICD-10 chapters) had small counts per unit time at subnational level. To reduce the noise introduced by sparse counts, we aggregated data by year. When aggregating time series data by a larger time interval, models will be fit to fewer data points, which may also cause an issue, such as increased uncertainty in estimates.

Our study has some limitations. The quality of mortality data varied by country and changed over time, as discussed in detail in the national-level study ^7^. To minimize the influence of data quality, each country collected and cleaned the data using standardized methodology in collaboration with the Pan American Health Organization (PAHO). We also controlled for changes in data quality by including the control cause of death in the model, assuming that changes in data quality affect both the outcome and the control. Our results, however, should be interpreted carefully. Regarding the data on PCV uptake, some municipalities in Brazil had unreliable data exceeding 100% with abrupt increases and decreases. We defined the income level of each municipality using the data in the year of PCV introduction, although the income level could change over time. For trend adjustment, we were only able to include one control cause of death (all-cause deaths other than those caused by respiratory conditions) due to data sparsity. In Peru, even common control causes of death only had 0–2 deaths per year in many regions. However, we believe that all-cause deaths were able to successfully adjust for some key trends because its estimated coefficient was different from zero in many regions.

One challenge with the type of analysis conducted here is that vaccine uptake data tend to be less reliable at local scales. Estimating both the numerator (number of children vaccinated) and denominator (number of children eligible for the vaccine) becomes more challenging at small spatial scales. Because the vaccine uptake data were measured with error, the estimates for the association with pneumonia deaths could be biased towards zero. This trend was clear especially among small low-income regions in Peru which were filtered out from the final analysis. Having higher quality data on vaccine uptake at a local level could facilitate robust estimates of vaccine impact in other countries and regions.

In conclusion, in three middle-income countries in South America, regions with higher PCV coverage experienced larger declines in pneumonia deaths, regardless of the income level. Focusing public health efforts on increasing the vaccine coverage could prevent additional childhood deaths.

## Data availability

### Underlying data

Zenodo: AHO_subnational 0.2; http://doi.org/10.5281/zenodo.4011097 ^14^.

Subfolder ‘Data’ contains the aggregated time series data used in this study. Data are also available at https://github.com/weinbergerlab/PAHO_subnational/tree/master/Data.

br.meso.v1.csv, co.meso.v1.csv, and pr.dept.inc.v1.csv include time series for subnational-level mortality data for Brazil, Colombia, and Peru, respectively. pr.cov.by.dept.inc.csv includes the PCV coverage data for Peru.

### Extended data

Figshare: OnlineSupplement.pdf; https://doi.org/10.6084/m9.figshare.12934901.v1 ^12^.

This file contains the following extended data:

- Supplementary methods
- Table S1. Average declines in all-cause pneumonia mortality among children aged 2–23 months expected with the 90% coverage of PCV by income level in Brazil and Peru using J12-18 coded as the primary cause of death as an outcome.

Figshare: Supplementary Figures; https://doi.org/10.6084/m9.figshare.12957887.v1 ^13^.

This file contains the following extended data:

- Figure S1. Uptake of the third dose of pneumococcal conjugate vaccines by subnational region in Brazil, Colombia, and Peru.
- Figure S2. Coverage of PCV third dose in the last year of the study period by income level in Brazil, Colombia, and Peru.
- Figure S3. Proportion of J12-J18 recorded as the primary cause of deaths among J12-J18 recorded as any causes of deaths in Brazil and Peru.

Analysis code available from: https://github.com/weinbergerlab/PAHO_subnational.

Archived analysis code at time of publication: http://doi.org/10.5281/zenodo.4011097 ^14^.

All data and code are available under the terms of the Creative Commons Attribution 4.0 International license (CC-BY 4.0).

## References

[1] Kupek E Vieira ILV: [Impact of PCV10 pneumococcal vaccine on mortality from pneumonia in children less than one year of age in Santa Catarina State, Brazil]. *Cad Saude Publica.* 2016;32(3):e00131414. 10.1590/0102-311X00131414 27049314

[2] Diaz J Terrazas S Bierrenbach AL: Effectiveness of the 10-Valent Pneumococcal Conjugate Vaccine (PCV-10) in Children in Chile: A Nested Case-Control Study Using Nationwide Pneumonia Morbidity and Mortality Surveillance Data. *PLoS One.* 2016;11(4):e0153141. 10.1371/journal.pone.0153141 27058873 PMC4825990

[3] Becker-Dreps S Amaya E Liu L: Changes in childhood pneumonia and infant mortality rates following introduction of the 13-valent pneumococcal conjugate vaccine in Nicaragua. *Pediatr Infect Dis J.* 2014;33(6):637–42. 10.1097/INF.0000000000000269 24445827

[4] Becker-Dreps S Blette B Briceño R: Changes in the incidence of pneumonia, bacterial meningitis, and infant mortality 5 years following introduction of the 13-valent pneumococcal conjugate vaccine in a "3+0" schedule. *PLoS One.* 2017;12(8):e0183348. 10.1371/journal.pone.0183348 28813518 PMC5558986

[5] Suarez V Michel F Toscano CM: Impact of pneumococcal conjugate vaccine in children morbidity and mortality in Peru: Time series analyses. *Vaccine.* 2016;34(39):4738–4743. 10.1016/j.vaccine.2016.07.027 27521230

[6] Schuck-Paim C Taylor RJ Alonso WJ: Effect of pneumococcal conjugate vaccine introduction on childhood pneumonia mortality in Brazil: a retrospective observational study. *Lancet Glob Health.* 2019;7(2):e249–e256. 10.1016/S2214-109X(18)30455-8 30683242 PMC6344339

[7] de Oliveira LH Shioda K Valenzuela MT: Declines in pneumonia mortality following the introduction of pneumococcal conjugate vaccines in Latin American and Caribbean countries. *Clin Infect Dis.* 2020;ciaa614. 10.1093/cid/ciaa614 32448889 PMC8516507

[8] Pan American Health Organization: Lineamientos básicos para el análisis de la mortalidad. Reference Source

[9] Mancero X: Medición de la pobreza por ingresos - Actualización metodológica y resultados.2019 Reference Source

[10] II. MARCO CONCEPTUAL. Accessed August 29, 2020. Reference Source

[11] Plummer M: rjags: Bayesian Graphical Models using MCMC. R package version 4-7.2018 Reference Source

[12] Shioda K de Oliveira L Toscano CM: OnlineSupplement.pdf. *figshare.*Online resource.2020 10.6084/m9.figshare.12934901.v1

[13] Shioda K Weinberger D Toscano CM: Supplementary Figures. *figshare.*Figure.2020 10.6084/m9.figshare.12957887.v1

[14] Shioda K Weinberger D: weinbergerlab/PAHO_subnational 0.2 (Version 0.2). *Zenodo.* 2020 10.5281/zenodo.4011097

