# Supplementary material for: Impact of pneumococcal conjugate vaccine uptake on childhood pneumonia mortality across income levels in Brazil, Colombia, and Peru
Source: Gates Open Res. 2020 Sep 22;4:136. [Version 1] doi: 10.12688/gatesopenres.13187.1 (PMC7780121; doi:10.12688/gatesopenres.13187.1)
Supplement: Peer review history

# Peer review: Reviewer response for version 1

**DOI:** 10.21956/gatesopenres.14392.r30085

**Published:** 2020-12-21

**Authors:** Edward Kim Mulholland

**Affiliations:** 1 Department of Infectious Disease Epidemiology, Department of Paediatrics, University of Melbourne, Melbourne, Vic, Australia; 2 Infectious Disease Epidemiology, London School of Hygiene and Tropical Medicine, London, UK

This is a very timely and appropriate piece of research.  In the past some (including this reviewer) have argued that PCVs have the potential to increase inequity in child survival unless high coverage is reached in the poorest communities.  This research addresses this issue in 3 countries of the Americas where quality mortality data are available at subnational level.

 In each country departments or states are categorized into 3 levels based on income data from other sources.  Then the pneumonia mortality rates were evaluated by income level and time.  To account for important differences in PCV coverage by income, the authors extrapolated to a hypothetical PCV coverage level of 90%.  This seems particularly important in Peru where PCV coverage ranged from 67% to 94% in the 3 strata.  Exactly how this extrapolation was done is unclear to this reviewer.  Logically the relationship between PCV coverage and mortality is non-linear as the unvaccinated will be at higher risk, regardless of income level, and this effect may be more acute at lower income levels. Figure 2 seems to show that this adjustment had little effect on the conclusions.

 Figure 3 which shows estimated mortality declines with PCV coverage is a bunch of straight lines with various data points on it.  What does this mean?

 On Page 5, “In Peru, this association was especially strong in low-income regions, while it was modest among high-income regions and not clear among medium-income regions.” This seems a polite way to put this.  The graph seems to show increasing mortality among most of the middle income regions. This probably reflects issues with data, but it is difficult to conclude that.  At least it should be discussed.

 As a reader it is a little difficult to really understand the paper without the basic pneumonia mortality data in the 3 strata against which the trends are being analysed.  All data are presented relative to the year of vaccine introduction, 2009, 2010 and 2008 for Brazil, Colombia and Peru respectively. The 3 countries have all seen substantial falls in child mortality over the period which predated PCV introduction, so the key question seems to be what was the impact of PCV, over-and-above the declines that were taking place. That would appear to be presented in Figure 1, which seems to show that there was no significant decline in any of the 3 countries, once non-pneumonia mortality trends were accounted for.

 Figure S3 is difficult to follow, probably because of the legend.

 In conclusion, as a reader I was reassured that PCV is associated with reduced child pneumonia mortality in most communities in the 3 countries, but this is quite in line with overall changes in child mortality in those countries so it is difficult to attribute the decline to PCV.

Is the work clearly and accurately presented and does it cite the current literature?

Partly

If applicable, is the statistical analysis and its interpretation appropriate?

I cannot comment. A qualified statistician is required.

Are all the source data underlying the results available to ensure full reproducibility?

Yes

Is the study design appropriate and is the work technically sound?

Partly

Are the conclusions drawn adequately supported by the results?

Partly

Are sufficient details of methods and analysis provided to allow replication by others?

Partly

Reviewer Expertise:

Infectious disease epidemiology; vaccinology, paediatrics

I confirm that I have read this submission and believe that I have an appropriate level of expertise to confirm that it is of an acceptable scientific standard, however I have significant reservations, as outlined above.

# Peer review: Reviewer response for version 1

**DOI:** 10.21956/gatesopenres.14392.r30069

**Published:** 2020-12-18

**Authors:** Robert Simon Heyderman

**Affiliations:** 1 NIHR Global Health Mucosal Pathogens Research Unit @UCL, University College London, London, UK

This study sets out to evaluate how the association between increasing vaccine uptake and population-level declines in death due to pneumonia in children 2-23 months varies by socioeconomic status in Brazil, Colombia, and Peru. The authors have recently published an evaluation of the population-level impact of PCVs against death due to pneumonia in children under 5 years of age. The value of such national level analyses is size but the datasets from Peru and Colombia are modest. The analysis is confounded by heterogeneity of vaccine programmes and falling pneumonia death rates before vaccine introduction. These data nonetheless would be a valuable addition to the literature.

 I have some suggestions that could improve the quality of the manuscript:

**TITLE:** The authors have specifically focused on children 2-23 months of age – this should be reflected in the title.

**INTRODUCTION:**

 Although it may seem obvious, the authors should introduce what is already known and why differences in pneumonia deaths related to socioeconomic status between and within countries may be expected.

**METHODS:**

1. This analysis is highly dependent on the coding of pneumonia episodes in each country, uniformity of practice between countries and over time. The reader is pointed to a WHO document on standardized data cleaning and quality control which is in Spanish. Some further detail should be provided. Have any checks been done to ascertain miscoding? Do the authors have any data to demonstrate the fidelity of the coding.
2. The authors state that income level was ascertain in “household surveys”. Were these undertaken by the national governments?
3. To provide a context for the data, the authors should state the approximate birth cohorts for each country and the total number of deaths during the same period.
4. To account for overdispersion, an observation-level random effect was included in the analysis. Is there evidence that this was successful?
5. Were permissions required to use this national data?

**RESULTS:**

1. Table 1 shows that the total number of J12-18 among children 2–23 months in Brazil coded as primary or non-primary causes of death is 16,303 4700 and the total number of J12-18 among children 2–23 months of age in the study period coded as primary cause of death is 35,259. Is this the correct way around?
2. The authors provide the weighted average of PCV third dose coverage in the last year of the study period in the main text but the rapidly of vaccine uptake and the difference between and within countries are best illustrated by Figure S1 which should be included in the main manuscript.
3. To understand trends, all-cause pneumonia has been adjusted for all-cause mortality other than respiratory illness but the underlying trend could be in infectious diseases only. What is the overall trend in non-pneumonia infectious diseases? Has this been used to adjust all-cause pneumonia?
4. Figure 3 is referred to in the statement “Higher uptake of the third dose of PCV was associated with additional declines in pneumonia deaths in most of the regions in all three countries”. An analysis should be provided to confirm what seems to be apparent visually.

**DISCUSSION:**

1. The statement, “The estimated decline in all-cause pneumonia deaths associated with increasing PCV uptake was similar across income levels in Brazil and Colombia, while there was a larger decline among low-income regions in Peru”, should include a statement about the uncertainty.
2. Limitations should include: the number of pneumonia deaths were relatively modest in Peru and Columbia; heterogeneity in schedules and vaccine valency which may be particularly relevant for younger ages; and the estimates of vaccine coverage.
3. The main conclusion is that “in three middle-income countries in South America, regions with higher PCV coverage experienced larger declines in pneumonia deaths”. This needs to be formally substantiated in the results (see above).

Is the work clearly and accurately presented and does it cite the current literature?

Partly

If applicable, is the statistical analysis and its interpretation appropriate?

I cannot comment. A qualified statistician is required.

Are all the source data underlying the results available to ensure full reproducibility?

Yes

Is the study design appropriate and is the work technically sound?

Yes

Are the conclusions drawn adequately supported by the results?

Partly

Are sufficient details of methods and analysis provided to allow replication by others?

Partly

Reviewer Expertise:

Microbial Pathogenesis; Mucosal Immunity; Vaccine Preventable Diseases

I confirm that I have read this submission and believe that I have an appropriate level of expertise to confirm that it is of an acceptable scientific standard, however I have significant reservations, as outlined above.

# Peer review: Reviewer response for version 1

**DOI:** 10.21956/gatesopenres.14392.r29638

**Published:** 2020-10-21

**Authors:** Tamara Pilishvili

**Affiliations:** 1 Respiratory Disease Branch, National Center for Immunizations and Respiratory Diseases, Centers for Disease Control and Prevention, Atlanta, GA, USA

This is an important study contributing to the body of literature documenting PCV impact on mortality. The authors apply complex analytic methods to evaluate association between increasing vaccine uptake and pneumonia-related deaths by socioeconomic factor. The paper is well written and methods are clearly explained.

 Specific comments:

- Declines in mortality are reported; however, for all estimates wide CrIs and all estimates cross the null value. Is the conclusion about the declines observed in all countries in the last year of study warranted given high level of uncertainty around the estimates? Perhaps the conclusion and corresponding statements in the results could be softened.
- How do the authors explain increases in mid-income regions in Peru and mid- and low-income regions in Brazil? Several lines for corresponding regions in Fig 3. suggest there were increases in deaths and the lines are above the dotted “null” line as vaccine coverage increases.
- Changes in coding practices over time could have contributed to observed trends. This should be included as a limitation.
- Unreliable PCV coverage data is mentioned in limitations without explanation of how this was resolved in the analysis (e.g. >100% coverage or unstable coverage estimates).
- Have any control “exposures” been evaluated, in addition to adjusting for “control cause of deaths”? If an association is found between control “exposures” (e.g. vaccine uptake other than PCV) that should not be associated with pneumonia-related deaths, this could indicate that the model is not adjusting for time-dependent confounders.

A couple of minor comments:

- Table 1. Number of deaths for Brazil appear to be flipped - numbers are higher for pneumonia as primary-only cause of death than for pneumonia as primary or secondary cause of death.
- Figure 1B interpretation in the text suggests that after adjusting for non-respiratory causes of death, the trends became “unclear”. From the figure, it appears that for most strata there was no longer a downward trend before or after PCV introduction.

Is the work clearly and accurately presented and does it cite the current literature?

Yes

If applicable, is the statistical analysis and its interpretation appropriate?

Partly

Are all the source data underlying the results available to ensure full reproducibility?

Yes

Is the study design appropriate and is the work technically sound?

Yes

Are the conclusions drawn adequately supported by the results?

Partly

Are sufficient details of methods and analysis provided to allow replication by others?

Yes

Reviewer Expertise:

NA

I confirm that I have read this submission and believe that I have an appropriate level of expertise to confirm that it is of an acceptable scientific standard.
